# New treatment options for metastatic HER2-low breast cancer: Consequences for histopathological diagnosis

**DOI:** 10.1007/s00292-022-01139-4

**Published:** 2022-12-06

**Authors:** Carsten Denkert, Annette Lebeau, Hans Ulrich Schildhaus, Christian Jackisch, Josef Rüschoff

**Affiliations:** 1 Institute of für Pathology, Philipps-University Marburg and University Hospital Marburg (UKGM), Baldingerstr. 1, 35043 Marburg, Germany; 2 Institute of Pathology, Universitätsklinikum Hamburg-Eppendorf (UKE), Hamburg, Germany; 3 Private Group Practice for Pathology, Lübeck, Germany; 4 Discovery Life Sciences, Kassel, Germany; 5 Pathologie Nordhessen, Kassel, Germany; 6 Department of Gynecology and Obstetrics, Sana Klinikum Offenbach GmbH, Offenbach, Germany

**Keywords:** Breast cancer, HER2, HER2-low, Antibody–drug conjugate, Immunohistochemistry, HER2-Gene, Immunkonjugate, Immunhistochemie, Zielgerichtete molekulare Therapie, Trastuzumab

## Abstract

The overexpression of HER2 in breast cancer is a classic example for molecular targeted therapy, and it has been shown that classical anti-HER2 therapeutics were only effective in patients with HER2 overexpressing tumors. Therefore, in recent decades, pathologists have been focused on the reliable identification of HER2 overexpressing tumors. Based on the results of recent clinical trials in metastatic breast cancer with antibody-drug conjugates (ADCs), this diagnostic strategy for evaluation of HER2 is currently changing. It has been shown that the ADC trastuzumab-deruxtecan is effective not only against tumors with classical HER2 overexpression, but also against HER2-low tumors. These clinical trial results lead to a paradigm shift in the treatment of patients whose tumours were previously classified as HER2 negative. In addition to the identification of HER2 (score 3+) overexpressing tumors, it is necessary to identify HER2-low expressing tumors (defined as an immunohistochemistry (IHC) score of 1+ or IHC2+ with negative in situ hybridization).

Due to the therapeutic consequences, it is important to quickly adapt the diagnostic workup and reporting to the new requirements. In addition, the new therapeutic options for anti-HER2 therapy lead to new challenges for standardization as well as to new scientific questions for the characterization of tumors with low HER2 expression.

## Introduction

Based on the results of clinical trials [18], trastuzumab was approved as a treatment option for HER2-positive metastatic breast cancer in the EU in 2000. In 2006, it was approved in the adjuvant setting. Subsequently, other anti-HER2 therapeutic strategies were established, including dual blockade with trastuzumab/pertuzumab and treatment with the antibody–drug conjugate (ADC) T‑DM1. Thus, therapeutic options include monotherapy with trastuzumab as well as dual blockade with anti-HER2-directed antibodies or tyrosine kinase inhibitors such as lapatinib or neratinib.

The main treatment concept in all previous therapies was that anti-HER-directed agents were only effective in HER2-positive tumors with strong overexpression at the protein level and/or *HER2* gene amplification. In the NSABP B47 trial [9] and in a translational investigation of the GeparQuattro trial [3], it was confirmed that there was no therapeutic benefit of trastuzumab in tumors without HER2 overexpression. Thus, for diagnostic pathology, there was a clearly defined diagnostic aim of reliable identification of those tumors with HER2 overexpression. The HER2 evaluation is based on guidelines initially established as part of the approval of trastuzumab by the U.S. Food and Drug Administration (FDA) [6] and subsequently adapted by the American Society of Clinical Oncology/College of American Pathologists (ASCO/CAP) in the HER2 Guidelines 2007 [32], 2013 [33], and 2018 [34]. In parallel, regular interlaboratory comparisons were established.

**Fig. 1:**
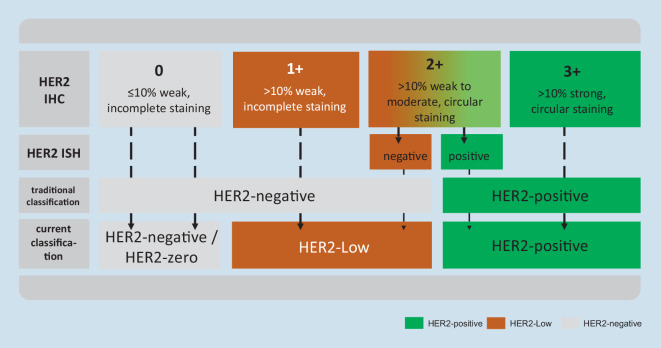
Strategy for HER2 assessment in breast cancer—overview of the current therapeutically relevant groups. *ISH* in situ hybridization

Based on new study results presented at ASCO 2022 and concurrently in the *New England Journal of Medicine* [14], it is now also necessary to identify and report tumors with low HER2 expression (HER2-low; defined as 1+ or 2+ with negative in situ hybridization [ISH]) and to establish a standardized reporting for this group of tumors (Fig. 1). In general, the evaluation categories defined in the current ASCO/CAP guideline are the still the basis for HER2 assessment. However, the test strategies and the wording in the histological report should be adapted in order to provide an optimal basis for clinical decisions.

## New therapeutic options—results of current clinical trials

Trastuzumab deruxtecan (DS-8201a; T‑DXd) is a novel ADC directed against HER2 [19]. The ADC technology is based on linking specific antibodies to a cytotoxic agent through a chemical compound (linker). The antibodies directed against HER2 specifically transport the cytotoxic agent to the tumor cell, where the toxin—in this case a topoisomerase inhibitor—is cleaved and internalized to exert its cytotoxic effect. Thus, systemic effects are minimized and side effects can be reduced. Trastuzumab deruxtecan is not only directly effective in strongly HER2-positive tumor cells, but also exerts its effect in tumor cells with HER2-low expression. Due to release of the toxin in the tumor tissue, there is an additional effect on neighboring cells (known as the “bystander killing effect”). An overview of selected current clinical studies on trastuzumab deruxtecan is presented in Table 1.

**Table 1:** Overview of selected clinical trials with trastuzumab deruxtecan

Trial	Tumor Type	Study design	Outcome	Reference
*Selected clinical trials in breast cancer*				
DESTINY-Breast01 Phase 2	HER2-positive metastatic breast cancer	Phase 2 trastuzumab deruxtecan after previous therapy with T‑DM1	61% response	Modi et al., NEJM 2020 [15]
DESTINY-Breast03	Metastatic HER2-positive breast cancer	Trastuzumab deruxtecan vs. T‑DM1	Positive study result PFS: 75.8% vs. 34.1% Overall response rate 79.7% vs. 34.2%	Cortes et al., NEJM 2022 [2]
DESTINY-Breast04	Metastatic HER2-low breast cancer (IHC1+ or 2+/SISHneg)	Trastuzumab deruxtecan (T-DXd) vs. physician’s choice chemotherapy (capecitabin, eribulin, gemcitabine, paclitaxel)	Positive study result (for details see text) The trial results are relevant for HER2 assessment in histopathological routine	Modi et al., NEJM 2022 [14]
Daisy study	Metastatic breast cancer (HER2-positive, HER2-low, and HER2 negative)	Phase 2 Trastuzumab deruxtecan	Response rates: HER2 3+: 71% HER2-low: 37.5% HER2-neg: 30.0%	Mosele et al. ESMO breast 2022 [16]
*Selected trials in other types of tumors*				
DESTINY-Gastric01	Advanced carcinoma of the stomach or gastroesophageal junction with classic definition of HER2 positivity, (3+ or 2+/FISH positive)	Phase 2 Trastuzumab deruxtecan (T-DXd) vs. chemotherapy	Objective response rate (ORR) and survival (OS) were significantly better than in the chemotherapy control arm (OS: 12.4 vs. 8.4 months)	Shitara et al., NEJM 2020 [26]
DESTINY-CRC-01	Colorectal carcinoma without *RAS*/*BRAF* mutations, classic HER2-positive and HER2-low positive	Phase 2 Trastuzumab deruxtecan	HER2 IHC3+ or IHC2+/ISH positive tumors: objective treatment response 45% Other results still pending	Siena et al. Lancet Oncol 2021 [27]
DESTINY-Lung01	Non-small cell lung cancer Activating* ERBB2* mutation (independent of HER2 expression and *ERBB2* amplification)	Phase 2 Trastuzumab deruxtecan	Response in 55%	Li et al., NEJM 2021 [12]

*IHC* immunohistochemistry, *ISH* in situ hybridization,* SISH *silver-enhanced ISH, *FISH *fluorescence ISH

Most relevant for the current extended clinical indication and pathological diagnosis is the DESTINY-Breast04 trial presented at ASCO 2022 [14]. In this trial, patients with metastatic HER2-low breast carcinoma who had already received one or two lines of chemotherapy were randomized 2:1 to receive either trastuzumab deruxtecan (T-DXd) or the investigator’s choice of chemotherapy (capecitabine, eribulin, gemcitabine, paclitaxel).

Median progression-free survival (PFS) in the hormone receptor positive (HR+) cohort was 10.1 months in the T‑DXd group vs. 5.4 months in the control group. The hazard ratio (HR) for PFS was 0.51 (95% confidence interval [CI] 0.40–0.64; *p* < 0.0001). There was a similar significant benefit in the overall population, with a median PFS of 9.9 months in the T‑DXd cohort and 5.1 months in the chemotherapy group. A significant advantage for T‑DXd was also seen for overall survival. In the HR+ group, median OS was 23.9 months with T‑DXd and 17.5 months with chemotherapy. In the overall cohort, median OS results were similarly favorable for the ADC (T-DXd 23.4 months, chemotherapy 16.8 months, HR 0.64; 95% CI 0.49–0.84; *p* = 0.001). The trial data formed the basis for FDA approval of trastuzumab deruxtecan in August 2022 [7].

The new therapeutic options have led to an increased proportion of patients with metastatic breast cancer who may benefit from anti-HER2 therapy: in addition to patients with classically strongly HER2-positive carcinomas (approximately 15%) [15], a large group of patients with HER2-low-expression tumors (approximately 50%, depending on the study cohort) might also benefit from anti-HER2 therapy [29].

## Principles of HER2-low scoring

It should be emphasized that starting with the pivotal trials for trastuzumab in the late 1990s, there has always been a classification of HER2 expression into four groups (0, 1+, 2+, 3+). The positive study results with T‑DXd in HER2-low breast carcinoma in the DESTINY-Breast04 study were obtained based on the current 2018 ASCO/CAP recommendations [34] for HER2 determination in breast cancer. Thus, the currently existing guidelines are also the basis for HER2-low assessment.

**Fig. 2:**
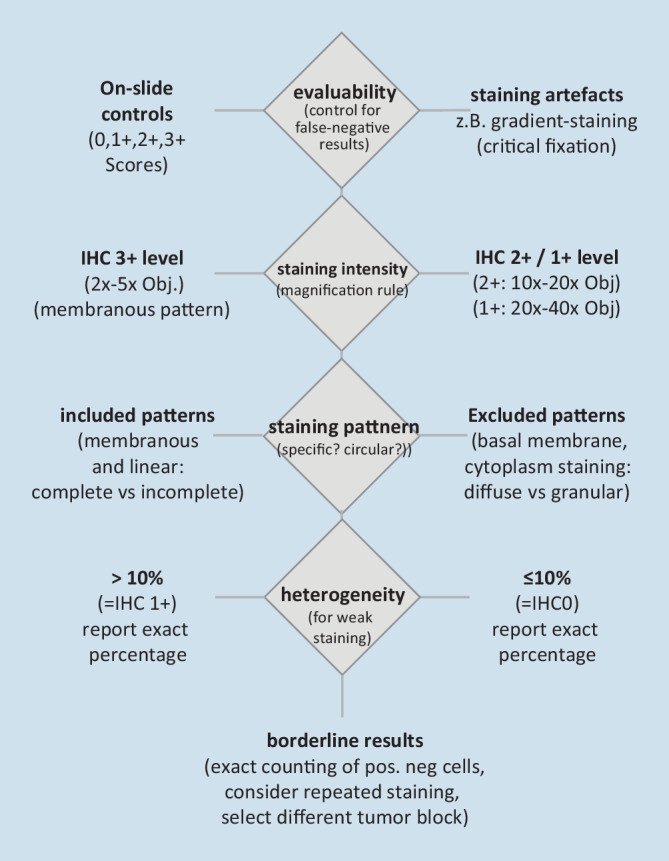
Workflow for standardized HER2 scoring using the magnification rule. *ICH* immunohistochemistry

Based on the new data, there is now a clinically relevant three-group assessment of HER2 expression that should be clearly stated in the histological report: HER2-negative (or HER2-zero; immunohistochemistry [IHC]0), HER2-low (IHC1+ or 2+/ISHneg), and HER2-positive (IHC3+ or 2+/ISHpos). As the new treatment options are currently available only for breast cancer, the diagnostic term “HER2-low” should currently only be used for this tumor type. Based on currently ongoing studies on the analysis of interobserver variance in HER2-low diagnostics, the following procedure is recommended for the assessment of immunohistochemical staining (Figs. 1 and 2):

### Step 1: Application of the “magnification rule” [23]

**Fig. 3:**
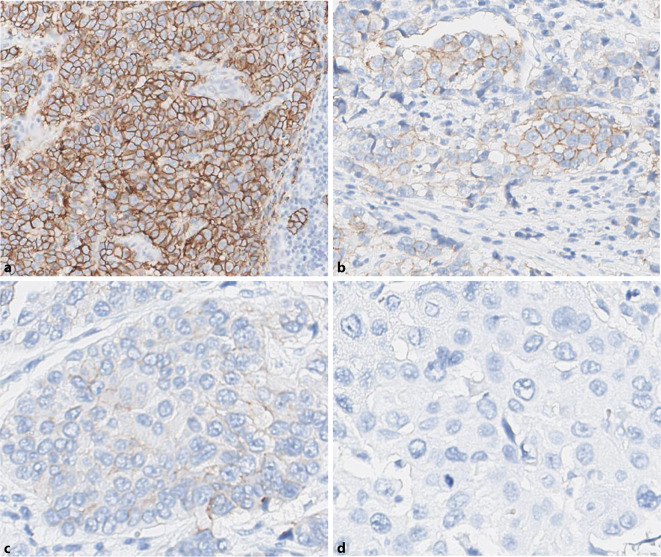
Examples of immunohistochemical determination of HER2 score using the magnification rule. **a** IHC3+: typical membranous staining pattern, already recognizable at low magnification (2–5 ×). **b** IHC2+: linear and circular membrane staining, clearly recognizable at 10–20 × magnification. **c** IHC1+: incomplete membrane staining, often detectable at 20 × magnification but 40 × magnification is usually also required to verify a specific membrane reaction. **d** IHC0: weak incomplete membrane staining in ≤ 10% of cells or complete absence of an immune response (4B5, VENTANA, Roche, Basel, Switzerland). *ICH *immunohistochemistry. (Note: For IHC2+ and moderate to strong but incomplete membrane staining, verification of gene amplification by in situ hybridization is required)

Here, the entire tumor area should be screened (Fig. 3) to determine at which magnification membranous HER2 expression can be detected.

- Strong HER2 staining (IHC3+) can be seen as clear membrane staining even when using a 2 × or 5 × objective.
- Moderate-strength HER2 (IHC2+) staining is typically seen as linear membrane staining at the cell–cell contact sites only when using a 10 × or 20 × objective.
- Weak (IHC1+) staining is typically not clearly identified as membrane staining until the 40 × objective is used.

Using this simple evaluation scheme as a first step allows a semiquantitative determination of the different staining intensities and reduces observer subjectivity.

### Step 2: Staining pattern—circularity of membrane staining

To clarify whether the score is IHC1+ or IHC2+, the second step is to evaluate the completeness of the membrane staining (circularity). Typically, complete circular staining of the cell membrane is detectable in IHC2+, whereas only partial staining is present in IHC1+. An exception may exist in gland-forming tumors, in which the apical region may be omitted from the staining (thus producing a U-shaped staining pattern to be interpreted as IHC2+).

**Fig. 4:**
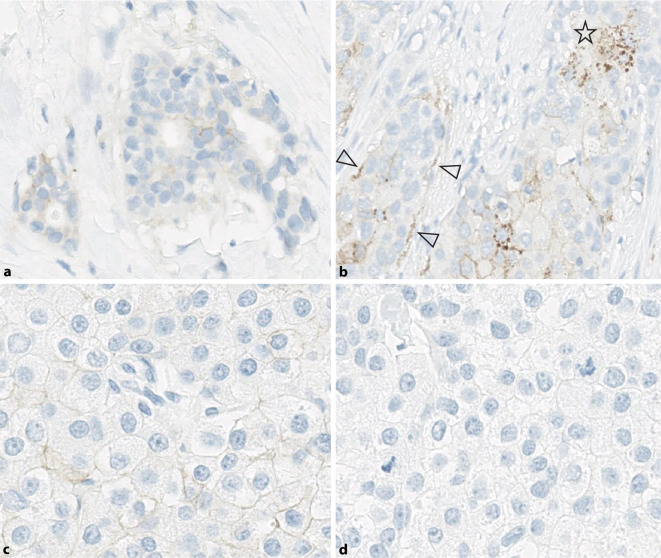
Typical pitfalls: staining patterns that might lead to misclassification. **a** Risk of false-negative scoring: case with just over 10% positive tumor cells underestimated, especially with weak membrane staining and tubular or single-cell (lobular) growth pattern. Recommendation: screen at 40 × magnification and count cells if necessary; correct score for **a**: IHC1+ (4B5, magnification 20 ×). **b** Risk of false-positive scoring. Staining in the basement membrane region (*arrowheads*) and granular cytoplasmic reactions (*asterisk*) should not be confused with the diagnostically relevant uniformly linear membrane staining (*bottom right of image*; correct score for **b** based on linear staining in the current image section: IHC1+; 4B5, magnification 20 ×). **c** Risk of false-positive, less frequently false-negative scoring: in tumors with a conventionally morphologically sharply demarcated cell membrane, it may be difficult to distinguish between specific membrane staining and native cell membrane. Recommendation: comparison with negative control staining (**d**), this results in a score of 1+ for **c** in the current image section by a very narrow margin, because only part of the membrane staining is specific when compared with the negative control (4B5, magnification 40 ×)

It is important to notice that aberrant staining patterns might exist, e.g., cytoplasmic staining or linear staining only in the basement membrane region. These staining patterns are not included (thus IHC0). Initial analyses of interobserver variability indicate that these staining patterns are the main cause of overscoring (e.g., IHC 1+ instead of 0, or 2+ instead of 1+; Fig. 4).

### Step 3: Percentage of tumor cells with HER2 expression

For an IHC score of 1+ (corresponding to HER2-low), at least 10% of tumor cells must have incomplete membrane staining, whereas for a score of 2+, at least 10% of tumor cells must have complete circular membrane staining. Thus, the third step is to determine the percentage of tumor cells defining the diagnostic score. It is useful to include this percentage in the histological report as well. Typical wording in the report could be, for example, “Evidence of weak, incompletely membranous staining in 25% of tumor cells, thus IHC1+.” In tumors with a heterogeneous distribution of staining, especially at the border between IHC1+ and IHC0, there exists a particular challenge, with the risk of both false-negative and (more rarely) false-positive assessment.

Cases with weak, incomplete staining just below the 10% cut-off are difficult to evaluate. Near the cut-off, especially in single-cell growth patterns (lobular, tubulolobular), the tumor cell count tends to be overestimated and the IHC score thus underestimated (Fig. 4). In case of doubt, tumor cells should be counted in the field of view (total number and positive cell count) and, if necessary, the difficult cases should be discussed with a colleague. Depending on the initial diagnostic situation, repeated staining on the same or a second tumor block (e.g., on the subsequent surgical specimen) may also be considered.

For tumors with a weak expression below the 10% cutoff, some authors also use the term HER2 “ultralow” [31]. This category, however, is not relevant for current clinical decisions and should therefore not currently be used in diagnostic reports to avoid unnecessary confusion.

## Practice of HER2-low testing

### Sample material

Tissue samples fixed in 10% neutral buffered formalin for between 6 h and 72 h and embedded in paraffin are suitable (FFPE samples) [32, 33]. In metastatic disease, reassessment of receptor status including HER2 is generally recommended regardless of primary HER2 status, because a change in HER2 status may occur during tumor progression. A change in HER2 status relative to the status at primary diagnosis can be expected in approximately 10% of metastatic tumors, with loss of expression observed in most cases (change from HER2-positive to HER2-negative) [25]. The first studies comparing HER2-low status showed discordance of HER2 expression (HER2-low versus HER2-zero) in up to 32% of cases, with a tendency for HER2-low expression to increase in advanced breast cancer [13, 28]. In the DESTINY-Breast04 study, HER2 determinations were performed on actual tumor biopsies or archival material from the primary tumors [14]. Therefore, if metastatic tissue is not available, FFPE material from the primary tumor may be used for assessment of HER2-low status. If quality-assured HER2 IHC was already performed as part of the primary diagnosis, a re-evaluation of the sections is recommended with special focus on HER2-low assessment. Analysis of multiple tissue samples from one tumor does not appear to be indicated based on the current results, as the available reports suggest that the majority of HER2-low carcinomas show a homogeneous staining pattern [14].

### Antibodies, technical platforms, regulatory framework

Identification of HER2-low status requires testing by HER2 IHC, thus testing by ISH alone is not a suitable diagnostic strategy. To ensure the validity and reproducibility of HER2 determination, it is recommended to use standardized assays according to the manufacturer’s instructions in the appropriate automated IHC staining systems.

In the context of DESTINY-Breast04, the VENTANA HER2/neu (4B5) assay (Roche, Basel, Switzerland) was used. In Europe, drug approval is generally not linked to specific diagnostic assays, i.e., other assays or primary antibodies may also be used as “laboratory-developed tests” (LDT). In the US, the FDA has typically approved defined assays for biomarker evaluation, including detection of HER2 expression and gene amplification [8]. This process is currently ongoing for the evaluation of HER2-low for establishing the indication for trastuzumab deruxtecan treatment.

In Germany, in addition to the 4B5 assay on the VENTANA BenchMark automated staining systems, the rabbit polyclonal antibody from DAKO/Agilent on the autostainer is one of the main test kits used (HercepTest for Automated Link Platforms; Agilent, Santa Clara, CA, USA). When the two test kits were compared in 500 breast carcinomas, the overall percent agreement was 73.5% (95% CI 69.1–77.0) [21]. The main reason for the discrepancies between the two assays was that tumors tended to be classified in higher HER2 categories with VENTANA 4B5 compared to the HercepTest.

Whether these results could be reproduced by other research groups and also apply to the new test assay HercepTest^TM^ mAb pharmDx (Dako Omnis, Agilent) or LDTs with monoclonal primary antibodies such as CB11, SP3, or EP3 is currently being evaluated. In a recent comparative study, the new HercepTest assay (GE001) tended to have a slightly higher HER2 category classification compared to 4B5, with an overall agreement of 83.7% [22].

However, the observed discrepancies highlight the need to run continuous quality assurance programs for evaluation of the immunohistochemical methods. In the future, this should not only include reliable identification of classically HER2-positive cancers, but also of HER2-low cancers. For this purpose, the use of on-slide controls using cell lines with defined HER2 staining patterns in the low range is recommended, as well as regular participation in round robin tests, offered, for example, by the German Quality Assurance Initiative Pathology (QuiP).

## T-DXd in other indications and in *HER2* mutations

The *ERBB2* gene encodes a receptor tyrosine kinase whose expression and activation play a role in different types of human cancers. Pathogenic activation can occur through gene amplification with consecutive overexpression, but also through pathogenic mutations. In addition to breast cancer, this also affects molecular subgroups of rectal cancer [30], gastric cancer [11], cholangiocellular cancer [1], and lung cancer [20]. Based on these results, the efficacy of T‑DXd is currently also being clinically investigated in additional tumor types.

In the phase II DESTINY-Gastric01 trial [26], patients with HER2-positive (3+ or 2+/ISH+) advanced cancer of the stomach or gastroesophageal junction were treated from the second line onward. The objective response rate (ORR) and overall survival (OS) were significantly better than in the chemotherapy control arm (OS 12.4 vs. 8.4 months).

There are also initial published trial results for colorectal cancer [27]. Tumors without *RAS*/*BRAF* mutations were treated in two cohorts with classical HER2 overexpression/amplification or with HER2-low expression. For the cohort with HER2 IHC3+ or IHC2+/ISH-positive tumors, an objective treatment response of more than 45% was reported. Further trial data are pending. In lung cancers with *HER2* mutations, partial response or complete tumor regression was observed in over 50% of patients [12]. Interestingly, the effect occurred with both typical kinase domain mutations (*ERBB2* exon 20) and with mutations affecting the extracellular domain, and also appeared to be independent of HER2 expression status and *ERBB2* gene amplification.

## Current challenges and scientific questions

Translational investigations in a cohort of 2310 patients with breast cancer from clinical trials of the German Breast Group [4] showed clinical and biological differences between HER2 completely negative (IHC0, HER2-zero) and HER2-low tumors. HER2-low tumors are more often hormone receptor positive, have a lower response rate to neoadjuvant chemotherapy in both the overall cohort and the HR-positive subcohort, and show an improved prognosis after neoadjuvant therapy in the HR-negative subgroup that did not respond to neoadjuvant therapy. Similar results, especially the positive correlation between HER2-low status and a positive HR status, were also shown in other studies [24].

Another open question is how low the HER2 status may become for trastuzumab deruxtecan therapy to still be effective. In the DAISY study [5], evidence for an effect in even HER2-negative (IHC0) tumors was shown: these tumors might have minimal HER2 expression below the detection level. However, it is not clear whether these findings can be confirmed in a prospective phase III study, so there is currently no relevance for histological diagnosis and treatment decisions in daily clinical practice.

An important focus for standardization of HER2 assessment is the extension of EQA trials to include HER2-low tumors. Current data from clinical trials suggest that there is room for improvement in this area. In a recent study [10], only 26% concordance among 18 pathologists was found when evaluating as 0 or 1+. However, it should be noted that the participating pathologists were blinded to the actual study objective, i.e., they were not told that concordance between IHC0 and IHC1+ would be evaluated. Thus, interpretation of existing interobserver comparisons must take into account that it has not been clinically relevant to reliably distinguish between IHC0 and IHC1 in the past. The two groups were therefore partially combined in histological reports as HER2-negative. Extended studies and analyses of HER2 expression patterns, including from the DESTINY-Breast 04 study, are currently ongoing.

It is expected that interobserver concordance will increase significantly when it is widely known that reliable differentiation of IHC0 and IHC1+ will provide new therapeutic options for patients. A similar effect was found for concordance of classical HER2 evaluation: in an analysis of HER2 assessment in GBG trials over 12 years in Germany [17], it was shown that since 2011, there has been 92% concordance for HER2 overexpression upon comparing local pathology assessment with central pathology. In contrast, in the years prior to 2006, there were much higher discrepancies, with 25–50% discrepant cases. This shows that diagnostic standardization and concordance for a biomarker increase once there is a treatment indication based on this biomarker. This has been observed for diagnosis of the HER2 3+ group; therefore, a similar improvement in concordance can be expected for the HER2-low group.

## Conclusions for clinical practice

Considering the results of the DESTINY-Breast04 study and the resulting new therapeutic options with antibody-drug conjugates directed against HER2, important conclusions arise for practical diagnostic pathology:

- A clear distinction between HER2 immunohistochemistry (IHC)0 and IHC1+ should be made in the histological report. The two groups should not be combined into a “HER2-negative” group in the report.
- The new group should be clearly identified in the pathology report. We recommend the use of the term “HER2-low.”
- The term “HER2-low” should currently only be used for breast cancer; the treatment option is currently only available for metastatic breast cancer. However, standardized evaluation with documentation of the individual IHC scores should be performed for all tumor types.
- Histological diagnosis is still based on the current ASCO/CAP guidelines for HER2 diagnosis. The evaluation of strongly HER2-positive tumors by immunohistochemistry (for IHC3+) or the combination of IHC and in situ hybridization (for IHC2+) also remain identical. The distinction between 0 and 1+ can only be made by IHC.
- For the evaluation of IHC, the standardized procedure described in this article (1: magnification rule; 2. staining pattern; 3. percentage) is recommended. The percentage of positive cells should be included in the diagnostic report. This is relevant for the differentiation between IHC0 (≤ 10% weak positive cells with incomplete membrane staining) and IHC1+ (> 10% weak positive cells with incomplete membrane staining). In this way, it is possible to directly identify which tumors are in the borderline area.
- The primary tumor or a re-biopsy of the metastasis can be used for the determination of HER2-low status; however, the current clinical guidelines recommend a re-biopsy with re-determination of the receptor status (including HER2) as standard in the case of tumor progression, if this is clinically possible.

## Declarations

For this article no studies with human participants or animals were performed by any of the authors. All studies mentioned were in accordance with the ethical standards indicated in each case.

The supplement containing this article is not sponsored by industry.
